# PVDF-TrFE-Based Stretchable Contact and Non-Contact Temperature Sensor for E-Skin Application

**DOI:** 10.3390/s20030623

**Published:** 2020-01-22

**Authors:** Bastien Marchiori, Simon Regal, Yanid Arango, Roger Delattre, Sylvain Blayac, Marc Ramuz

**Affiliations:** Flexible Electronics Department, Ecole Nationale Supérieure des Mines CMP-EMSE, MOC, 880 avenue de Mimet, 13541 Gardanne, France; bastien.marchiori@emse.fr (B.M.); simon.regal@emse.fr (S.R.); yanid.arango@mines-stetienne.fr (Y.A.); roger.delattre@emse.fr (R.D.); blayac@emse.fr (S.B.)

**Keywords:** stretchable electronic, artificial skin, infrared sensing, temperature sensing, organic electronic

## Abstract

Development of stretchable electronics has been driven by key applications such as electronics skin for robotic or prosthetic. Mimicking skin functionalities imposes at a minimal level: stretchability, pressure, and temperature sensing capabilities. While the research on pressure sensors for artificial skin is extensive, stretchable temperature sensors remain less explored. In this work, a stretchable temperature and infrared sensor has been developed on a polydimethylsiloxane substrate. The sensor is based on poly(vinylidene fluoride-trifluoroethylene) (PVDF-TrFE) as a pyroelectric material. This material is sandwiched between two electrodes. The first one consists of aluminium serpentines, covered by gold in order to get electrical contact and maximum stretchability. The second one is based on poly(3,4-ethylenedioxythiophene):poly(styrenesulfonate) (PEDOT:PSS) that has shown good electrical compatibility with PVDF-TrFE and provides the stretchability of the top electrode. Without poling the PVDF-TrFE, sensor has shown a sensitivity of around 7 pF.°C^−1^ up to 35% strain without any change in its behaviour. Then, taking advantage on infrared absorption of PEDOT:PSS, a poled device has shown a pyroelectric peak of 13 mV to an infrared illumination of 5 mW at 830 nm. This stretchable device valuably allows an electronic skin (e-skin) use for contact and more importantly non-contact thermal sensing.

## 1. Introduction

The development of flexible and stretchable electronics has opened the possibilities to many new outstanding applications. Compared with rigid, hard conventional electronic systems, making devices few micrometers thin on flexible polymeric substrates such as polyethylene naphthalate [1] or polyimide [2] allows them to be conformal. For wearable electronics, very thin flexible devices have the ability to conform to the skin, and stretchable devices can go even further, following any deformation. Indeed, stretchable electronic systems can be stretched, compressed, bent, and deformed into arbitrary shapes without electrical or mechanical failure within the circuits. Stretchable electronics concern electrical and electronic circuits that are elastically or inelastically stretchable by more than a few percent while retaining function [3]. 

For application onto the skin or for mimicking it, this property is mandatory since the skin can stretch up to 15% and is constantly moving [4]. These stretchable devices are thus particularly suitable for developing sensing or actuating devices distributed directly on human skin. Through the engineering of a matrix of sensors, it would be possible to reproduce the sensing properties of the skin for robotic or prosthetic applications. Main functions of the skin include barrier protection, temperature, and tactile / pressure sensing. Sensing is carried out by a variety of recognition elements that transduce pressure, vibration, touch, stretch, strain, temperature, pain, and proprioception [5]. The integration of these sensors in a large area is commonly known as electronic skin (e-skin). 

To conform and deform according to the body, there are several features requested for the electronic system. An important need is to develop stretchable interconnections onto stretchable substrate in order to transmit signals from the sensors. One approach consists in using intrinsically stretchable metallic interconnections such carbon nanotubes or silver nanowires mixed with or deposited on a stretchable matrix, typically polydimethylsiloxane (PDMS) [6,7]. Despite high stretchability (up to 1000%), conductivity of such composite remains low (less than 10^4^ S.cm^−1^ reported at 80% strain [7,8]). Another strategy for interconnections relies on the use of bulk metallic materials with appropriate serpentine design that will permit deformation similarly to an in-plane string. The use of serpentines is more adapted to thick materials (>1 µm) because they can be deformed themselves [9,10,11]. Thinner materials tend to follow the deformation of the substrate, thus, using a pre-strain is more efficient [10,11].

Stretchability of 100% is more than sufficient for e-skin application, and conductivity is similar to bulk metal (10^5^ S.cm^−1^). Active sensing area could be either fully stretchable or based on rigid materials immersed into the stretchable interconnection/substrate matrix. The previous method—so-called island architecture—use more established and reliable sensing components, and recorded signal is less sensitive to mechanical deformation [1,12,13,14]. 

An objective of e-skin in prosthetics is to reproduce capabilities of the skin on a robotic arm for instance. The use of pressure sensors for giving the feeling of touch is an active topic of research, and the transmission of the response of the sensors to the brain has been studied [15]. However, integrating temperature sensing capabilities remains a challenge and particularly for non-contact sensing. 

For applications in temperature sensing, Kim et al., have developed a complete e-skin with pressure, temperature sensors based on a silicon PN junction [14]. This system is accurate, but the silicon is intrinsically not stretchable and needs a more complicated process of fabrication compared to the solution presented here. Based on the temperature coefficient of resistance, Webb et al., have experimented a resistance temperature sensor device that can be attached to the skin [16]. This solution is also adapted to contact temperature measurement and is simpler than the Si-based sensor because it relies only on a metallic line but is difficult to miniaturize. Among all the kind of temperature measurements, another possibility is to use pyroelectric materials. The material contains preferentially oriented dipoles at a stable state at a certain temperature. When the temperature increases, the dipoles tend to relax, leading to the creation of current through the change of polarization [17]. This device has the advantage of being very sensitive to the change in temperature; a simple measure the voltage induced is linked to the change of temperature. To go further and measure also the temperature in a non-contact mode, it is very common to put a black body on top of the pyroelectric element in order to make an infrared (IR) sensor.

The polyvinylidene fluoride (PVDF) is a material widely used for its high piezoelectric properties, first reported by Kawai et al. [18], and also for its pyroelectric properties [19]. Depending on the process used for the deposition of the PVDF, the crystallization leads to different structures, but only one presents the crystalline structure suitable for pyroelectric properties (β-phase) [20]. Nevertheless, the PVDF does not crystallize naturally in the good phase; it needs to be annealed and largely stretched [21]. In order to allow direct crystallization of the β-phase from the solvated state, it is possible to use poly(vinylidene fluoride-trifluoroethylene) (PVDF-TrFE) copolymers [20,22]. Once it has crystallized, it is possible to pole the copolymer in order to create the pyroelectric properties by applying a high electric field across the layer [20,23,24].

In addition, this material possesses a peak of absorption for wavelengths after 7 µm. From Wien’s displacement law, a maximum of emission below 7 µm corresponds to temperature below 140 °C. So, the PVDF is well adapted for detection of the human body for example [25]. For the detection of far IR radiations, one solution is to use special conditions of evaporation to deposit porous gold known as black-gold [26,27]. For near IR, another possibility is to use an electrode that has absorption properties in the desired wavelength band. Previous work has shown the feasibility of IR sensor with PVDF in the far IR [28,29]. Pecora et al., have also shown the operation of a flexible PVDF-TrFE pyroelectric sensor under absorption of a specific radiation (632 nm) [30]. This work offers the possibility to go further and demonstrates a stretchable temperature sensor with IR sensing capabilities. 

In this study, a stretchable device able to probe temperature as well as IR illumination in order to do contact and non-contact thermal sensing has been fabricated. The device is made out of two electrodes with PVDF-TrFE in between, within a PDMS matrix. The first electrode consists of bulk aluminium covered with gold to improve electrical contact. Indeed, the aluminium has a natural oxide layer which reduces conductivity. The interconnection lines have been designed in a serpentine shape in order to provide 70% stretchability of the line itself as already demonstrated by our group [31]. Then, the second electrode has been studied in order to provide stretchability, not only maintaining a conductivity but also high absorption in the near IR range of stretchable poly(3,4-ethylenedioxythiophene):poly(styrenesulfonate) (PEDOT:PSS) formulation. Response of the device to IR illumination is described. Then, response of the sensor regarding the temperature for different mechanical strain applied is also studied. 

## 2. Materials and Methods

*Interconnection fabrication*. The laser cutting of metallic film was made using LPKF Protolaser S equipment (Garbsen, Germany). The radiation was 1064 nm, frequency 75 MHz, power 10 W. The diameter of the beam was 25 µm. The aluminium tape was laminated on a glass slide (7.62 cm × 2.54 cm) using a thermal release double-sided Nitto (Osaka, Japan) 120 °C RevAlpha tape in between. The PDMS was spin coated at 300 rpm for a thickness of 350 µm and then cured at 70 °C for at least 3 h. Then, the RevAlpha tape was released on a hotplate at 135 °C for 1 min.

*Active material deposition.* The PVDF-TrFE (from PolyK Technologies, Philipsburg, PA, USA) was spin-coated at 1500 rpm, from a solution of 10% in Methyl Ethyl Ketone for a thickness of 3 µm through a polyimide (Kapton from Dupont, Wilmington, DE, USA) mask. The device was then annealed at 150 °C for 30 min. Gold (100 nm) was evaporated on the PVDF-TrFE through a shadow to get a proper electrical contact when the top PEDOT:PSS electrode was laminated on top.

*Top electrode fabrication.* The conducting polymer formulation consisted of PEDOT:PSS (Heraeus, Clevios PH 1000, Hanau, Germany), with ethylene glycol (Sigma-Aldrich, St. Louis, MO, USA) 0.25 mL for 1 mL PEDOT:PSS solution, 4, dodecylbenzenesulfonic acid (DBSA) (0.5 μL.mL^−1^), (3-glycidyloxypropyl)trimethoxysilane (GOPS) (10 mg.mL^−1^), and Capstone^®^ FS-30 (Wilmington, DE, USA). The solution was drop-casted on a PDMS sample after a mild plasma treatment at 25 W without oxygen using a PE-100 plasma System (Plasmatech, Carson City, NV, USA). This PEDOT:PSS on PDMS was cut around the drop and laminated on top of the PVDF-TrFE/gold stack and then, PDMS was spin coated at 300 rpm to ensure sealing of the device.

*Poling of the devices.* The device was poled with an aluminium electrode laminated and pressed as a top electrode and the bottom electrode was the one on which the PVDF-TrFE layer was deposited. The setup was provided by the company IRLYNX (Meylan, France) and made out of a sinusoidal generator with an amplifier. The devices were poled at 700 Vpp with 10 periods of signal at a frequency of 0.5 Hz. This voltage was the limit for the poling of the device. Higher voltage resulted in burning of the device at electrode contact.

*Mechanical/electrical characterization.* The real-time measurement of the capacitance was done with an Agilent 4263B LCR meter, calibrated with a 47 pF capacitance. The system is highly resistive, so the capacitance was extracted by measuring the parallel capacitance. Real-time measurement of the voltage was done using a Keithley 2636A. The real-time measurement of the temperature used as reference was done by placing a KTY81-210 temperature sensor (NXP Semiconductors N.V., Eindhoven, The Netherlands) on the device. The non-contact heat source was an electronic heat gun with an adjustable and controllable temperature. The output was set up at 80 °C and placed in the direction of the sample at 10 cm. For static measurement of the capacitance as a function of the temperature, a hot plate was used, and the corresponding temperature was taken from the temperature sensor after 5 min stabilization required for the hotplate to have a stable temperature. Stretching tests were performed on a custom-designed XY motion table with motors from ETEL (Môtiers, Switzerland). The device under test was clamped between two 3D-printed plastic jaws. The speed of the motor was set at 100 μm.s^−1^. Measurements, displacement, and data collection were performed simultaneously using a homemade LabVIEW script.

## 3. Results and Discussion

### 3.1. Design of the Sensor

**Figure 1:**
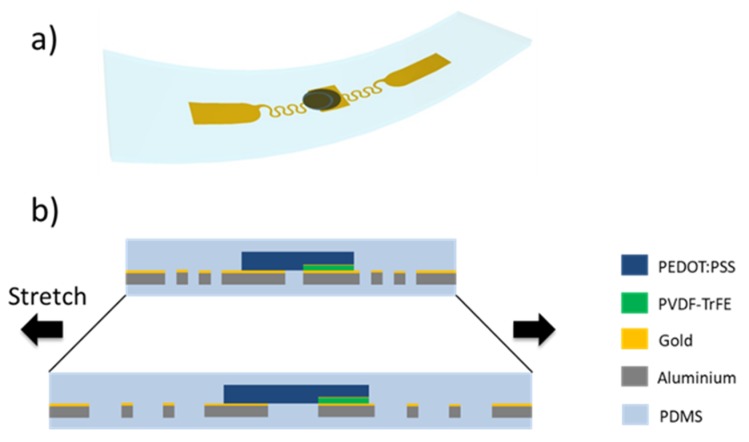
(**a**) 3D schematic of the sensor. (**b**) Cross section view. Poly(vinylidene fluoride-trifluoroethylene) (PVDF-TrFE) is sandwiched between two electrodes. Bottom electrode is a metal disk made of aluminum tape covered with gold. The top one consists of laminated poly(3,4-ethylenedioxythiophene):poly(styrenesulfonate) (PEDOT:PSS). Good electrical contact is ensured by an evaporation of gold on top of the PVDF-TrFE. The stretchable PEDOT:PSS is used to interconnect both electrodes.

The design of this IR sensor takes the full advantage of the 50 µm thick metallic interconnections by using an island approach. A schematic of the device is displayed in Figure 1. The interconnections were connected in the middle to a round-shape island of 5 mm diameter and on the other side to a pad used to take the signal. A layer of PVDF-TrFE was spin-coated, constituting the bottom electrode. The top electrode, made of modified PEDOT: PSS mixture [6,31] allowing to enforce the stretchability between the PVDF-TrDE active area and the electrode. This stretchable buffer PEDOT:PSS layer permits a planar configuration of interconnections with extended stretchability and IR absorption properties. A thermal infrared sensor is constructed around the absorption of a black body material (PEDOT:PSS here) on a thermal sensor (PVDF-TrFE). The material absorbs infrared radiations and produces heat that is detected by the thermal sensing layer through pyroelectricity. 

The stretchability of the device is both ensured by the horseshoe shaped interconnections and by the use of optimised PEDOT:PSS [31,32,33]. The formulation of PEDOT:PSS uses a fluoro-surfactant to enhance its stretchability up to 40% (Figure S1). The designed interconnections have the advantage of being stretchable by more than 70% while keeping a stable resistance. Previous work has demonstrated that the horseshoe shape of the line allows the line to release the constrains by deformation out of the plane of the stretch [31]. Fabrication process is easy and fast with the use of a laser for patterning. The proper integrity of the PVDF-TrFE sensing area was achieved by capping it with a rigid metallic round, itself part of the interconnection.

### 3.2. Characterization of the Device under Temperature Change

Two types of temperature sensing mode are identified. The first comes from the change of capacitance of a poled or non-poled device. This mode allows absolute measurement of temperature upon appropriate calibration. The second mode is based on pyroelectric properties of the device. Once poled, devices release a voltage that can be measured and is correlated to temperature change. It is important to note that this mode of operation measure transient changes of temperature [34].

**Figure 2:**
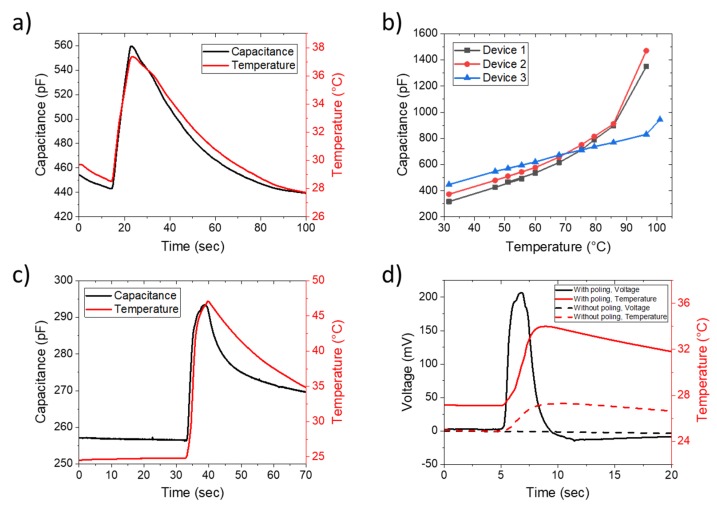
(**a**,**b**) Electrical characterization of non-poled sensors with temperature change. (**a**) Capacitance response for temperature change, (**b**) capacitance as a function of the temperature for 3 devices. (**c**,**d**) Characterization of a device after poling. (**c**) Change of the capacitance and (**d**) voltage generated due to the change of temperature. Baseline correction from −200 mV to 0 V is applied.

Figure 2a presents capacitance and temperature as a function of time for a non-stretched configuration. The temperature was measured using an external temperature sensor placed beside the device and recorded at the same time than the capacitance. Changes of capacitance followed closely the change of temperature from the temperature sensor, which validates the temperature sensing through capacitance measurement.

The capacitance as a function of temperature from ambient temperature to 100 °C is displayed in Figure 2b for three identical devices. Linear response to temperature below 60 °C with an average sensitivity of 7.45 pF.°C^−1^ is observed for devices 2 and 3. Then, above 60 °C, capacitance starts to deviate from the trend; and above 100 °C, capacitance is not measurable. Device 1 shows a higher initial capacitance, but a sensitivity reduced to 5.95 pF.°C^−1^. Therefore, without poling, it is possible to use the device as a temperature sensor by simply measuring its capacitance.

Devices show a linear response to temperature change without poling through the measurement of the capacitance. However, measurement of capacitance is not direct. It requires a more complicated circuit to extract the value compared to voltage, only obtainable after poling. Initially, distribution of dipoles in PVFD-TrFE are randomly distributed; there is no natural piezoelectric properties. Poling aligns the dipoles and is carried out through applying a high electric field. Figure 2c,d presents graphs of the characterization after poling of the device by applying 700 V in between bottom and top electrodes (more details available in Methods Section). Figure 2c shows changes in capacitance as a function of temperature. Sensitivity of the capacitance decreases to 1.6 pF.°C^−1^. Voltage created due to the poling, called pyroelectricity, is displayed in Figure 2d. Voltage sensed through the electrodes is stable and presents a peak of 200 mV when the temperature increases by 7 °C by the use of heat gun. This voltage represented the derivative of the temperature change. From the baseline, voltage increases when temperature increases, and vice versa, it decreases when temperature decreases. The peak of voltage corresponds to the maximum increase in temperature while the signal passes by 0 V when the temperature is decreasing. During cooling, voltage remains slightly lower than the baseline because the temperature is decreasing slowly.

### 3.3. Characterization of the Device under Illumination

The poled device can also be used for non-contact temperature sensing. For IR temperature sensor, absorption of the top electrode was studied. Then, device under illumination of IR radiations was monitored. 

**Figure 3:**
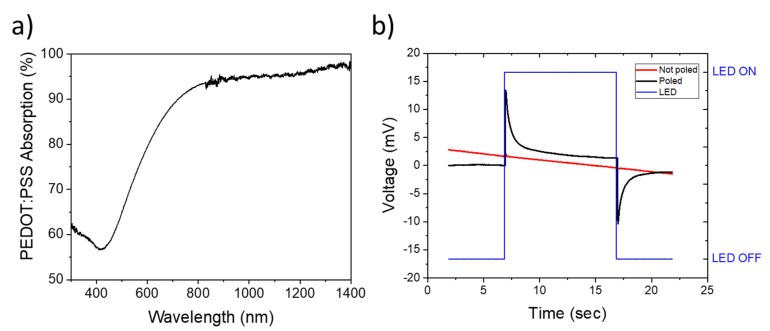
(**a**) Absorption spectrum of a PEDOT:PSS thick layer. (**b**) Device under IR illumination at 830 nm. The PEDOT:PSS absorbs the radiations, creating a local heat source that induces a voltage for poled devices. Baseline correction from −700 mV to 0 V is applied. The non-poled device did not create any voltage variation.

Gold is a commonly used electrode, which shows a high electrical conductivity but presents poor IR absorption properties. Moreover, as mentioned before, the PVDF-TrFE does not absorb IR wavelengths below 7 µm. A replacement electrode must have absorption in the IR to absorb a radiation of LED, low resistivity to carry the signal, and finally stretchability to be integrated for e-skin application. The properties of PEDOT:PSS have already shown high stretchability and conductivity with the addition of a fluorosurfactant [31,32]. In addition, a thick layer of PEDOT:PSS absorbs radiations between 600 nm and 1400 nm (Figure 3a). PDMS is a good choice of substrate since it does not absorb in these wavelengths (absorption spectrum available in SI, Figure S2).

The response of the sensor to the illumination under an LED at a wavelength of 830 nm is displayed in Figure 3b. The intensity of the illumination was measured at 0.3 mW.mm^−2^, which gives a power of 5.7 mW on the 19 mm^2^ surface of the sensor. LED was kept on for 10 s and then turned off. Non-poled devices do not show any voltage response to the illumination. Poled devices, on the contrary, display a very sharp signal change. As soon as the LED is turned on, a peak of voltage is measured. This peak represents a dynamic response, which describes the rapid change of temperature. The peak amplitude is 13 mV, which is 10 times higher than flexible device reported in the literature for similar energy [30]. There is also a reversed peak when LED is turned off, with amplitude of −12 mV, so similar amplitude. This sharp peak showed that when the LED is turned off, dissipation is almost instantaneous.

### 3.4. Characterization of the Device under Stretch

**Figure 4:**
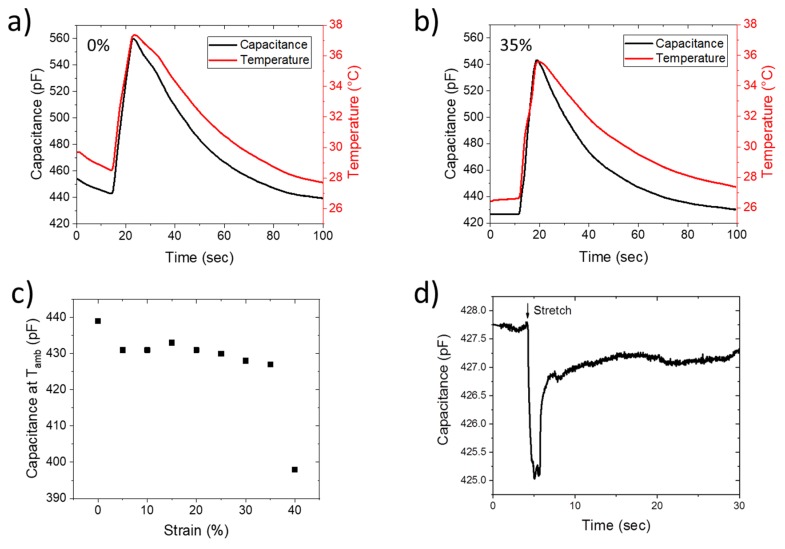
Stretching of a non-poled device for temperature sensing. (**a**,**b**) Response of the sensor to an increase of temperature at 0 and 35% stretch. (**c**) Capacitance at ambient temperature as a function of the strain. (**d**) Capacitance drop at ambient temperature of a device stretched from 25% to 35%.

Characterization of the capacitance as a function of temperature is displayed at different strains in Figure 4. The device has the same behaviour with temperature change at 0% stretch (Figure 4a) and at 35% (Figure 4b). It means that the change of resistance due to the stretch of PEDOT:PSS layer has no impact on the capacitance measurement. The measurement of capacitance of a non-poled device at the ambient temperature as a function of the deformation is displayed in Figure 4c. Capacitance decreases by only 3% between 0 and 35% strain. Then, it decreases from 430 pF to 400 pF between 35% and 40%. Further measurement of the capacitance after 40% was then impossible to read because of rupture of PEDOT:PSS layer.

The graph of change of capacitance of a non-poled device, from 25% to 35% strain at a constant temperature, is displayed in Figure 4d. There is a transient drop of capacitance of 3 pF when the device is stretched by 5%. This is due to the piezoelectric properties of the PVDF-TrFE. The pressure and the strain have the same effect than the temperature on its response. Then, when the displacement is finished, capacitance almost come back to its initial value, losing 0.8 pF, which is equivalent to an error of less than 0.1 °C for this sensor. This loss could indicate that, despite the presence of rigid metallic layer, PVDF-TrFE is stretched and is then slightly changing its spatial arrangement. 

## 4. Conclusions

This work demonstrates for the first time the use of thick metallic serpentines and stretchable PEDOT:PSS in an island configuration for fabrication of a non-contact temperature sensor. The non-stretchable PVDF-TrFE film is placed on the interconnections to avoid deformations in this area, keeping stable electrical characteristics. Final devices permit not only direct contact temperature but also IR sensing capabilities for non-contact temperature sensing. The sensor had an absolute temperature sensing sensitivity of 7 pF.°C^−1^ at no strain. No change in its performance is noticed up to 35% strain. Sensing of IR radiations is possible after poling without strain. Regarding results in the literature, optimisation is expected through an improved poling process in order to have better sensitivity, particularly regarding IR radiations. Other techniques have been developed and can be implemented in this device to extend its stretchability. Cai et al., have worked on the rigidification of specific areas of the substrates to avoid deformation [35].

The integration of this device in a matrix is the next step of this work. The resulting sensing layer can be the base for the design of a complete artificial skin. By using other PVDF pixels for pressure sensing, with only piezoelectric properties, a decorrelation between pressure and temperature would be attainable. Such artificial skin would provide full sensorial rehabilitation, mimicking all the capabilities of the skin.

## Supplementary Materials

The following are available online at https://www.mdpi.com/1424-8220/20/3/623/s1. Figure S1, resistance of PEDOT:PSS on PDMS with applied strain; Figure S2, absorption spectrum of PDMS. Figure S3, voltage sensed through the device before and after 10% strain.
