# Supplementary material for: PVDF-TrFE-Based Stretchable Contact and Non-Contact Temperature Sensor for E-Skin Application
Source: Sensors (Basel). 2020 Jan 22;20(3):623. doi: 10.3390/s20030623 (PMC7037770; doi:10.3390/s20030623)
Supplement: Supplementary file 1 [file sensors-20-00623-s001.pdf]

sensors

MDPI

Supplementary

PVDF-TrFE Based Stretchable Contact and Non-Contact Temperature Sensor for E-Skin Application

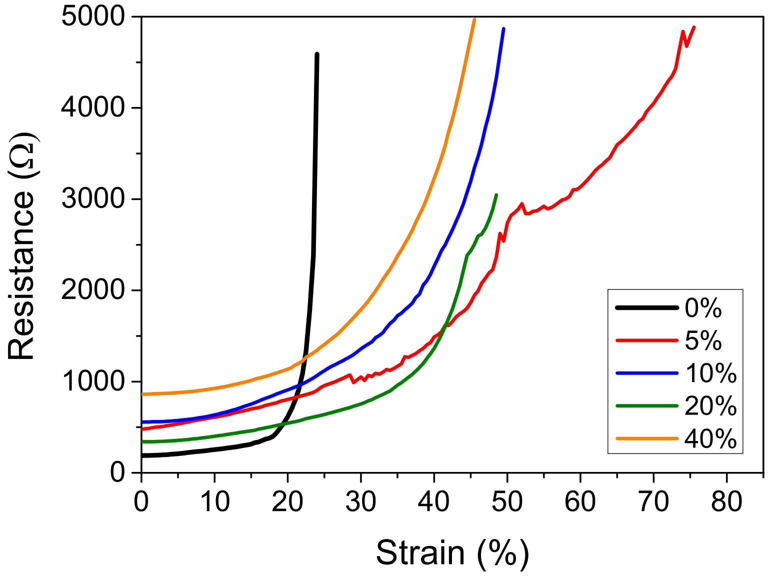

Figure S1. Optimisation of the formulation for the PEDOT:PSS layer, spin-coated on a PDMS substrate. Resistance as a function of the strain for different vol% of Capstone®. The sample is 3.81 mm by 2.54 mm and spin-coated at 1500 rpm for 30 seconds. Reproduced from [31].

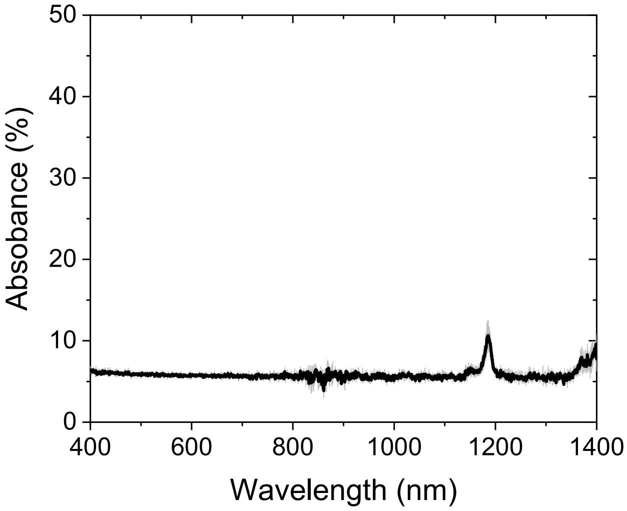

Figure S2. Average absorbance spectrum for PDMS versus the wavelength measured with a UV 2600 spectrophotometer (Shimadzu, Japon) equipped with the ISR-2600Plus integrating sphere (Shimadzu, Japon). The data are extracted from 3 samples. The grey surface shows the standard deviation. This

material has a low absorbance peak at 1200 nm. However it absorbs less than 10% of the emitted light from 400 to 1150 nm, and is therefore an appropriate choice as a substrate.

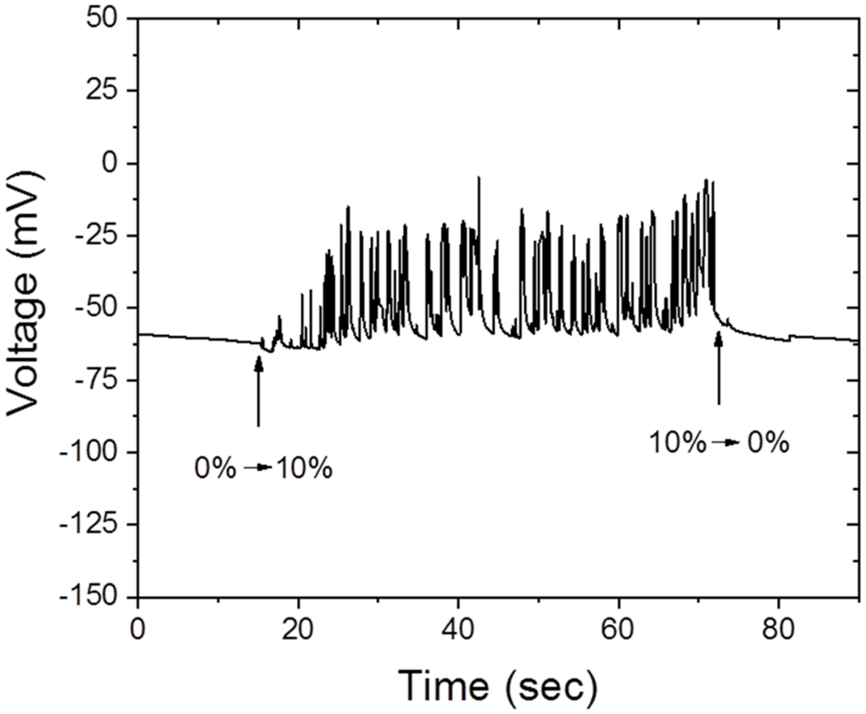

Figure S3. Voltage sensed through the device before and after 10% strain.
